# Immunological profiles associated with distinct parasitemic states in volunteers undergoing malaria challenge in Gabon

**DOI:** 10.1038/s41598-022-17725-8

**Published:** 2022-08-03

**Authors:** Mikhael D. Manurung, Sanne E. de Jong, Yvonne Kruize, Yoanne D. Mouwenda, Madeleine Eunice Betouke Ongwe, Yabo Josiane Honkpehedji, Jeannot Frézus Zinsou, Jean Claude Dejon-Agobe, Stephen L. Hoffman, Peter G. Kremsner, Ayola Akim Adegnika, Rolf Fendel, Benjamin Mordmüller, Meta Roestenberg, Bertrand Lell, Maria Yazdanbakhsh

**Affiliations:** 1 Department of Parasitology, Leiden University Center for Infectious Diseases (LU-CID), Leiden University Medical Center, Albinusdreef 2, 2333 ZA Leiden, The Netherlands; 2 Centre de Recherches Médicales de Lambaréné (CERMEL), Lambaréné, Gabon; 3 Institut de Recherches en Ecologie Tropicale, CENAREST, Libreville, Gabon; 4 Fondation Pour La Recherche Scientifique, 72 BP45 Cotonou, Bénin; 5 Center of Tropical Medicine and Travel Medicine, Department of Infectious Diseases, Amsterdam University Medical Center, Amsterdam, The Netherlands; 6 Sanaria Inc, Rockville, MD USA; 7 Institute of Tropical Medicine, University of Tübingen, Tubingen, Germany; 8 German Center for Infection Research, Partner Site Tübingen, Tübingen, Germany; 9 Division of Infectious Diseases and Tropical Medicine, Department of Medicine I, Medical University of Vienna, Vienna, Austria; 10 Present Address: Radboud University Medical Center, Nijmegen, The Netherlands

**Keywords:** Malaria, Malaria

## Abstract

Controlled human malaria infection (CHMI) using cryopreserved non-attenuated Plasmodium falciparum sporozoites (PfSPZ) offers a unique opportunity to investigate naturally acquired immunity (NAI). By analyzing blood samples from 5 malaria-naïve European and 20 African adults with lifelong exposure to malaria, before, 5, and 11 days after direct venous inoculation (DVI) with Sanaria^R^ PfSPZ Challenge, we assessed the immunological patterns associated with control of microscopic and submicroscopic parasitemia. All (5/5) European individuals developed parasitemia as defined by thick blood smear (TBS), but 40% (8/20) of the African individuals controlled their parasitemia, and therefore remained thick blood smear-negative (TBS^−^ Africans). In the TBS^−^ Africans, we observed higher baseline frequencies of CD4^+^ T cells producing interferon-gamma (IFNγ) that significantly decreased 5 days after PfSPZ DVI. The TBS^−^ Africans, which represent individuals with either very strong and rapid blood-stage immunity or with immunity to liver stages, were stratified into subjects with sub-microscopic parasitemia (TBS^-^PCR^+^) or those with possibly sterilizing immunity (TBS^−^PCR^−^). Higher frequencies of IFNγ^+^TNF^+^CD8^+^ γδ T cells at baseline, which later decreased within five days after PfSPZ DVI, were associated with those who remained TBS^−^PCR^−^. These findings suggest that naturally acquired immunity is characterized by different cell types that show varying strengths of malaria parasite control. While the high frequencies of antigen responsive IFNγ^+^CD4^+^ T cells in peripheral blood keep the blood-stage parasites to a sub-microscopic level, it is the IFNγ^+^TNF^+^CD8^+^ γδ T cells that are associated with either immunity to the liver-stage, or rapid elimination of blood-stage parasites.

## Introduction

Malaria remains a major global health challenge^1^. To control and eradicate the disease, a highly effective vaccine is needed. So far, vaccines tested in malaria-endemic regions have shown relatively low efficacy, which might in part be due to pre-exposure to malaria or other endemic infections^2–4^. Naturally acquired immunity has been demonstrated in endemic regions through controlled human malaria infection (CHMI) using injection of cryopreserved nonattenuated Plasmodium falciparum (Pf) sporozoites (PfSPZ) into volunteers with lifelong exposure to malaria parasites^5^. A better understanding of this naturally acquired immunity (NAI) might inform vaccine design for malaria-exposed individuals.

CHMI, owing to the defined onset and dose of infection, allows precise tracking of parasite emergence from the liver by microscopy and real-time PCR. Combined with immunological assays, CHMI could help distinguish whether a change in an immune feature is either a marker of exposure or protective immunity. Immune response to malaria involves both cellular^6–8^ and humoral^9,10^ immunity in various tissues to act throughout the malaria life cycle^11^. CD4^+^ T cells play important roles in activating other immune cells, such as NK cells^12^, or B cells^13^ through secretion of cytokines. CD8^+^ T cells are considered vital in protection against liver-stage malaria for their surveillance of the liver and subsequent IFNγ production to help infected hepatocytes in eliminating the parasites^14^. γδ T cells are important for inducing the aforementioned CD8^+^ T cell response^15^ and direct killing of blood-stage parasites through the production of various immune mediators such as IFNγ or TNF^16^. To disentangle this complex immunoregulatory network against malaria, an in-depth and broad immune profiling is needed.

Previously, we have performed systems analysis of immune response after direct venous inoculation (DVI) with non-attenuated PfSPZ in Africans with lifelong residence in a malaria-endemic area^17^. Individuals who remained thick blood smear (TBS) negative (TBS^−^ Africans) after PfSPZ DVI were characterized by, among other features, more IFNγ^+^CD4^+^ T cells at baseline^17^. Among the TBS^−^ Africans, several volunteers remained parasite negative even with qPCR (TBS^−^PCR^−^), a more sensitive assay, thus suggesting either liver-stage sterilizing immunity or rapid and strong control of blood-stage parasitemia. These groups have now been compared to identify immunological correlates associated with varying degrees of parasite control in individuals with lifelong exposure to malaria that might contribute to blood and liver-stage NAI.

## Materials and methods

### CHMI trial

**Figure 1:**
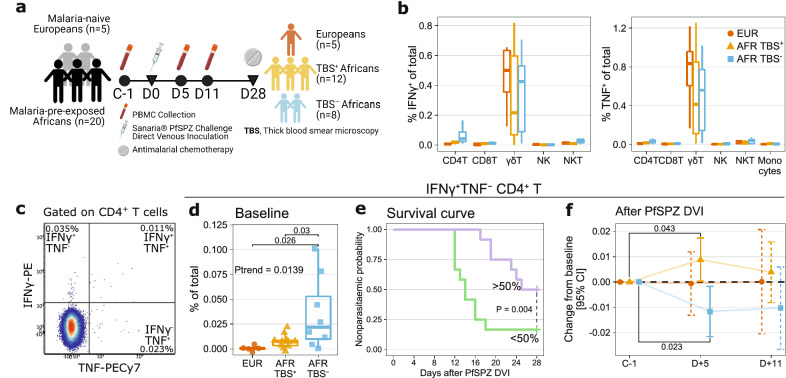
Higher baseline and early decrease in the frequencies of IFNγ-producing CD4^+^ T cells are associated with parasitaemia control in lifelong malaria-exposed individuals. **(a)** The LaCHMI study design and parasitological outcome of PfSPZ DVI with Sanaria^R^ PfSPZ Challenge. This figure is created with BioRender.com. (**b**) Frequencies of cells producing IFNγ or TNF upon PfRBC stimulation at baseline (C-1) in Europeans, TBS^+^ and TBS^−^ Africans. The boxplots show the median, 1st, and 3rd quartiles and the whiskers extend to the maximum/minimum of the respective groups, no further than 1.5 × the IQR. (**c**) Representative flow cytometry plot of IFNγ and TNF gating on CD4^+^ T cells. (**d-f**) IFNγ^+^TNF^-^CD4^+^ T cells at baseline and after PfSPZ DVI. (**d**) The frequency of IFNγ^+^TNF^−^CD4^+^ T cells at baseline. All data points are added to the box plots. P-values for the pairwise comparisons of the frequencies were performed using Tukey’s honestly significant difference (HSD) test. P-value for testing linear trend (P_trend_) was obtained from a linear model with orthogonal polynomial contrast. (**e**) Survival curve showing time until parasitemia according to the baseline frequency of IFNγ^+^TNF^−^CD4^+^ T cells. The frequency was split on the median to create two groups of individuals, a top > 50% (purple) and a bottom < 50% (green). This grouping was then used to compare the survival distributions of the groups using the log-rank test. (**f**) Changes in the frequencies of IFNγ^+^TNF^−^CD4^+^ T cells after PfSPZ DVI. The frequencies on five (D + 5) and eleven (D + 11) days after PfSPZ DVI were compared against the baseline using linear mixed models to obtain the estimates and corresponding 95% confidence intervals (CI) as well as P-values. The estimates and intervals were visualized using error bars with either solid lines if statistically significant (P < 0.05) or dashed lines if otherwise. P-values for significant comparisons are shown.

Samples analyzed in this study were taken from volunteers of a CHMI trial that was conducted by the Centre de Recherches Médicales de Lambaréné (CERMEL) in Lambaréné, Gabon, in August 2014 (Fig. 1a)^5^. The study was approved by the Gabonese National Ethics Committee (*Comité National d’Ethique de la Recherche*) and was conducted under the US FDA Investigational New Drug application. Before enrollment, written informed consent was obtained from volunteers. The study followed the principles of the Declaration of Helsinki in its sixth revision as well as the “International Council for Harmonization of Technical Requirements for Pharmaceuticals for Human Use–Good Clinical Practice (ICH-GCP)” guidelines. The safety of participants was supervised by an independent safety review committee. The study is registered at ClinicalTrials.gov under NCT02237586 (11/09/2014).

Five malaria-naïve European adults and 20 lifelong malaria-exposed African adults participated in the trial (Supplementary Table S1). Volunteers received a regimen of 12-hourly 300 mg clindamycin for 5 days to radically cure possible *Plasmodium* infection. Two days after the last clindamycin dose, volunteers were inoculated with 3200 non-attenuated PfSPZ (Sanaria^R^ PfSPZ Challenge). PfSPZ Challenge consists of aseptic, purified, infectious PfSPZ, strain NF54, produced and cryopreserved according to good manufacturing practices^18^. Volunteers were visited daily from day 5 after inoculation until treatment for blood sampling and to record symptoms. From day 5 onwards, TBS and quantitative PCR tests were performed daily to detect malaria parasites. Treatment with artemether-lumefantrine was administered to the European individuals upon detection of parasitemia by TBS. Treatment of the African individuals was initiated if either parasitemia was accompanied by symptoms typical of malaria, or irrespective of symptoms if there was parasitemia above 1000 parasites/μL, or at the end of the study at day 28 in those not previously treated.

### PBMC isolation and cryopreservation

Details of all materials and reagents used for this study are listed in Supplementary Table S2. Heparinised blood was diluted 1:2 with HBSS (100 U/mL penicillin G sodium and 100 ug/mL streptomycin), followed by the addition of approximately 10 mL 1.077 Ficoll and isolation of PBMCs. PBMCs were washed twice in HBSS and then cryopreserved in 20% fetal bovine serum (FBS) and 10% dimethyl sulfoxide (DMSO) in RPMI-1640 medium supplemented with 1 mM pyruvate, 2 mM l-glutamine, penicillin G, and streptomycin. The cells were placed overnight in a Nalgene Mr. Frosty Freezing Container (Thermo Scientific, Waltham, MA, USA) at – 80 °C before transfer to a liquid nitrogen container. The cryopreserved PBMCs were shipped in a liquid nitrogen dry vapor shipper from Lambaréné, Gabon, to Leiden, the Netherlands, for subsequent analysis.

### Cell culture

Cryopreserved PBMCs were thawed with 50% FCS/RPMI medium at 37^O^C. The median of cell recovery was 75% with 98.5% viability. Cells were rested overnight at 37 °C under 5% CO_2_ with 1 × 10^6^ cells/mL in cRPMI. On the next day, cells were brought into a 96-well U-bottomed plate (Corning) with 5 × 10^5^ cells/well, for a 24-h stimulation with either 5 × 10^5^ *P. falciparum*-infected red blood cells (PfRBC), 5 × 10^5^ uninfected red blood cells (uRBC), 200 ng/mL Staphylococcal enterotoxin B (SEB), or cRPMI. Intact PfRBC was obtained through purifying NF54 asexual-stage cultures using Percoll density gradient, followed by cryopreservation in 15% glycerol/PBS as described previously^19^; mock-cultured uRBC was obtained similarly. Brefeldin-A (10 μg/mL) was added for the final 22 h. After stimulation, PBMCs were stained with Aqua live-dead marker (Invitrogen™) for 30 min at room temperature and then fixed with 1.9% formaldehyde solution (Sigma). Afterward, cells were cryopreserved in 10% DMSO/20% FCS in RPMI and then stored at – 80 °C until analysis.

### Flow cytometry antibody staining and measurement

The fixed, cryopreserved cells were thawed and then stained in 96-wells V-bottom microplates with 50 µL of flow cytometry antibody mixture (Supplementary Table S3) diluted in eBioscience™ permeabilization buffer (ThermoFisher) with 1% human Fc-receptor blocker (eBioscience) at 4 °C for 30 min. Pooled cells were split equally for use as FMO controls. A median of 141,800 live, single cells was obtained. PBMCs were acquired with BD LSR-Fortessa X-20 SORP and BD FACSDiva 8.0.1. CST setup beads (BD Biosciences) and single-stained compensation beads (BD™ CompBead) were used to set up the flow cytometer and compensation matrix, respectively.

### Flow cytometry data analysis

Flow cytometry data were analyzed with FlowJo version 10.4.1 (TreeStar). The gating strategy is shown in Supplementary Fig. S2. For the gating of cytokine-producing cells, gates were initially set on FMO controls and then further adjusted according to the uRBC background response for each donor before finally applying the gate to samples stimulated with PfRBC.

For the unsupervised analysis of cytokine-producing CD4^+^ T and γδ T cells, those producing any IFNγ or TNF were exported as FCS files. The FCS files were then read using flowCore R/Bioconductor package^20^. The cytokine-producing cells were clustered using spectral clustering as implemented in Spectrum R package and visualized using UMAP as implemented in uwot R package^21^. Heatmap was made using ComplexHeatmap R/Bioconductor package^22^.

### Protein microarray

Protein microarray experiments and analyses were performed as previously described^23,24^. The microarray consists of 262 *P. falciparum* proteins representing 228 unique antigens expressed using an *Escherichia coli* lysate in vitro expression system and spotted on a 16-pad ONCYTE AVID slide, representing 228 important *P. falciparum* antigens known to frequently provide a positive signal when tested with serum from those with sterile and naturally acquired immunity against the parasite. Study serum samples from the baseline time point (C-1), as well as European control serum samples, were diluted 1:50 in 0.05 × Super G Blocking Buffer (Grace Bio-Labs) containing 10% *E. coli* lysate (GenScript) and incubated for 30 min on a shaker at room temperature. Meanwhile, microarray slides were rehydrated using 0.05 × Super G Blocking buffer at room temperature. Rehydration buffer was subsequently removed and samples added onto the slides. Arrays were incubated overnight at 4 °C on a shaker (180 r.p.m.). Serum samples were removed the following day and microarrays were washed using 1 × TBST buffer (Grace Bio-Labs). Secondary antibodies were then applied at a dilution of 1:200 and incubated for 2 h at room temperature on the shaker, followed by another washing step and a 1-h incubation in a 1:250 dilution of Qdot585 Streptavidin Conjugate. After a final washing step, slides were dried by centrifugation at 500* g* for 10 min. Slide images were taken using the ArrayCAM Imaging System (Grace Bio-Labs) and the ArrayCAM 400-S Microarray Imager Software v.2.2.

Microarray data were analyzed in R statistical software package v.3.6.2. All images were manually checked for any noise signal. Each antigen spot signal was corrected for local background reactivity by applying a normal-exponential convolution model using the RMA-75 algorithm for parameter estimation (available in the limma package v.3.28.14)^25^. Data were log_2_-transformed and further normalized by subtraction of the median signal intensity of mock expression spots on the particular array to correct for background activity of antibodies binding to *E. coli* lysate. After log_2_ transformation, data were normally distributed.

### *Plasmodium* gene expression data mining

To investigate the expression of genes encoding differentially reactive *Plasmodium* proteins across the *Pf* life cycle, we utilize the publicly available *Plasmodium* informatics resources, PlasmoDB (release 52, 20 May 2021)^26^. We mined for the expression of genes encoding RH5, MSP5, PHAX, STARP, EBA140, and PF3D7_1360400 in sporozoites harvested from mosquito salivary glands and asexual blood stage using the “mosquito or cultured sporozoites and blood-stage transcriptome (NF54)” RNA-Seq data set. Gene expression abundance is in transcripts per million (TPM) unit.

### Statistical analysis

Data analysis was performed with R version 4.0.2^27^ and RStudio version 1.0.143. The tidyverse R package was used to import, wrangle, and visualize data^28^. For the analysis of the frequencies of cytokine-producing cells, background responses to uRBC were subtracted from the paired PfRBC-stimulated sample. All statistical tests in this study were two-tailed with a significance level set at *P* < 0.05. For pairwise comparisons of baseline cell frequencies and *Pf* protein microarray data, Tukey’s honestly significant difference (HSD) test from the PMCMRplus R package was used, unless mentioned otherwise. To analyze the association between cytokine-producing cell frequencies and time-to-parasitemia (defined with either TBS microscopy or PCR), the log-rank test from the survival R package was used. The survival curve adjusted for covariates was generated by the survminer R package. For the analyses of the changes in cell frequencies after PfSPZ DVI, we used a linear mixed-effects model with heterogeneous variance, random-intercept, and unstructured covariance matrix using the nlme R package. Cell frequencies on day five (D + 5) and eleven (D + 11) after PfSPZ DVI were compared against the baseline (C-1) to obtain the statistical significance of the absolute change in frequencies over time. The estimated mean and 95% confidence interval (CI) of the change was obtained using the emmeans R package.

## Results

### Immune responses associated with the control of parasitemia

After PfSPZ DVI, all European (5/5) and 12 of 20 African individuals (60%) developed parasitemia detectable by TBS microscopy (TBS^+^) within the 28-day study period (Fig. 1a, Supplementary Fig. S1a, and Supplementary Table S1). The remaining eight (40%) African individuals remained TBS-negative for the whole study period, indicating varying degrees of naturally acquired immunity (NAI) among individuals with lifelong exposure to malaria. To investigate correlates of NAI against *P. falciparum*, we analyzed frequencies of immune cells producing IFNγ or TNF upon in vitro stimulation not only at baseline but also over time after PfSPZ DVI. In addition, cytokine-producing immune cells associated with NAI at baseline were correlated with antibody reactivity to *Plasmodium* proteins to provide mechanistic insights.

At baseline (C-1), γδ T and CD4^+^ T cells constituted the majority of cells producing IFNγ or TNF upon PfRBC stimulation among malaria pre-exposed Africans (Fig. 1b, Supplementary Fig. S3). The average frequency of γδ T cells, which have an innate response to PfRBC, that produced IFNγ or TNF tended to be higher in Europeans than in Africans, although not statistically significant (IFNγ^+^ γδ T mean ± SD, 0.84 ± 0.39 vs. 0.49 ± 0.41, t-test P = 0.37; TNF^+^ γδ T mean ± SD, 0.50 ± 0.22 vs 0.39 ± 0.28, t-test P = 0.28). We then analyzed the frequencies of CD4^+^ T cells co-producing IFNγ and TNF or only one of these cytokines (Fig. 1c). The frequency of IFNγ^+^TNF^−^CD4^+^ T cells was higher in TBS^−^ Africans compared to both TBS^+^ Africans (P = 0.03) and Europeans (P = 0.026) (Fig. 1d). Regarding other cytokine-producing CD4^+^ T cell subsets, we observed a significant increasing trend in the frequencies of IFNγ^+^TNF^+^CD4^+^ T cells, which was the lowest in Europeans and the highest in TBS^−^ Africans (P = 0.015; Supplementary Fig. S4). Survival analysis using the time to microscopic parasitemia showed that higher frequency of IFNγ^+^TNF^−^CD4^+^ T cells was associated with a higher probability of remaining TBS^−^ (Fig. 1e). Following PfSPZ DVI, the frequencies of IFNγ^+^TNF^−^CD4^+^ T cells significantly decreased early on day five (D + 5) in TBS^−^ Africans, whereas a significant increase was observed in the TBS^+^ Africans (Fig. 1f). Unsupervised analysis of cytokine-producing CD4^+^ T cells revealed two effector memory (EM, CD27^+^CD45RA^-^) clusters that showed the highest baseline frequencies in TBS^-^ Africans. However, no significant change in frequencies following PfSPZ DVI was observed (Supplementary Fig. S5). Thus, a higher baseline frequency and early decrease of IFNγ^+^TNF^−^CD4^+^ T cells were associated with resistance against parasitemia as detected by thick blood smear positivity.

**Figure 2:**
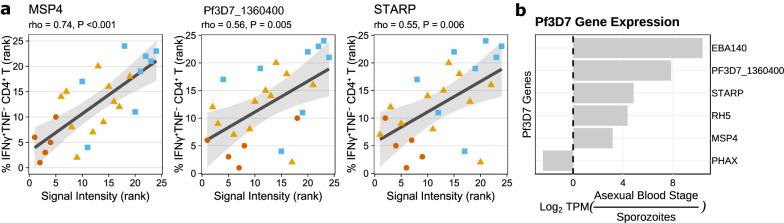
IFNγ-producing CD4^+^ T cells are positively correlated with antibodies against antigens highly expressed in malaria asexual blood stage. (**a**) Spearman correlations between frequencies of IFNγ^+^TNF^−^CD4^+^ T cells and antibody reactivity to Plasmodium protein microarray. All data points are added and the color of the points indicates the grouping used in Fig. 1. The black line and shaded gray area represent linear fit (y ~ x) on rank-transformed values and 95% confidence intervals, respectively. (**b**) Log_2_ ratio of gene expression (in transcript per million; TPM) encoding *Plasmodium* protein in malaria asexual blood-stage over malaria mosquito sporozoites. Genes were selected based on the association between levels of antibodies against the encoded protein and NAI^17^.

To further investigate the functional relevance of CD4^+^ T cells producing IFNγ^+^ in resistance against parasitemia, we correlated the frequencies of IFNγ^+^TNF^−^CD4^+^ T with antibody reactivity data to *Plasmodium* proteins that distinguish TBS^+^ and TBS^-^ Africans^17^. IFNγ-producing CD4^+^ T cells were positively correlated with MSP4 (rho = 0.74, P < 0.001), Pf3D7_1360400 (rho = 0.56, P = 0.005), and STARP (rho = 0.55, P = 0.006; Fig. 2a). Notably, the expression of genes encoding these proteins was consistently higher in the asexual blood-stage parasites compared to sporozoites (Fig. 2b). Therefore, IFNγ-producing CD4^+^ T cells are significantly correlated with antibody reactivities to *Plasmodium* proteins encoded in the blood-stage parasites.

**Figure 3:**
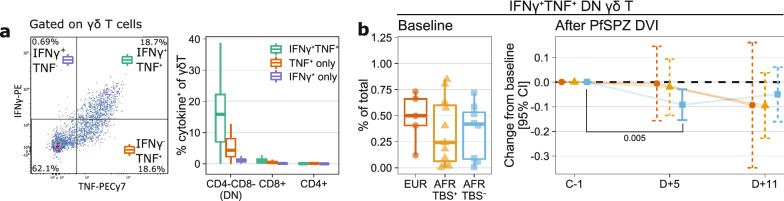
Early decrease in the frequencies of IFNγ^+^TNF^+^DN γδ T is associated with parasite control. (**a**) Representative flow cytometry plots of IFNγ and TNF gating on γδ T cells (left panel) and boxplots of frequencies of cytokine-producing γδ T subsets (CD4^−^CD8^−^ or DN, CD4^+^, CD8^+^) relative to total γδ T cells, irrespective of groups or time points (right panel). (**b**) Frequencies of IFNγ^+^TNF^+^DN γδ T cells at baseline (left panel) and after PfSPZ DVI (right panel). The boxplots show the median, 1st, and 3rd quartiles and the whiskers extend to the maximum/minimum of the respective groups, no further than 1.5 × the IQR. The frequencies on five (D + 5) and eleven (D + 11) days after PfSPZ DVI were compared against the baseline using linear mixed models to obtain the estimates and corresponding 95% confidence intervals (CI) as well as P-values. The estimates and intervals were visualized using error bars with either solid lines if statistically significant (P < 0.05) or dashed lines if otherwise. P-values for significant comparisons are shown.

We then investigated the frequencies of cytokine-producing γδ T cells at baseline and following PfSPZ DVI. We observed that most γδ T subsets co-produce IFNγ and TNF and that CD4/CD8 double-negative (DN) γδ T cells constituted the majority of cytokine-producing γδ T cells after PfRBC stimulation (Fig. 3a). The baseline frequency of IFNγ^+^TNF^+^DN γδ T cells was not significantly different among the groups (Fig. 3b, left panel). Following PfSPZ DVI, however, we observed a significant decrease in frequencies early on day five in TBS^−^ Africans (Fig. 3b, right panel).

Taken together, CD4^+^ T cells producing IFNγ, with or without TNF co-production, are associated with parasite control in lifelong malaria-exposed individuals. A potential mechanism by which IFNγ-producing CD4^+^ T cells orchestrate parasite control is highlighted by its correlation with antibody reactivities to blood-stage proteins. Regarding DN γδ T cells co-producing IFNγ and TNF, although the baseline frequencies were comparable among the groups, an early decrease within 5 days following PfSPZ DVI was observed in those remaining TBS^−^.

### CD8^+^ γδ T cells in individuals remaining PCR^−^ after PfSPZ DVI

**Figure 4:**
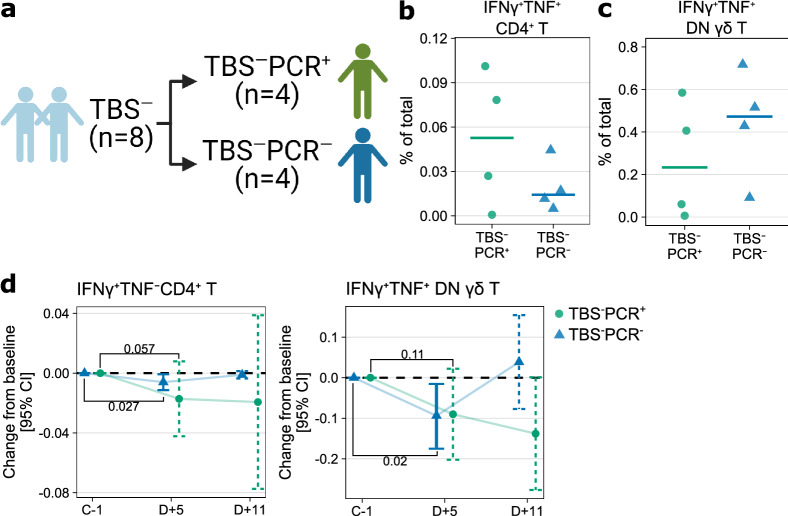
Cytokine-producing cell subsets associated with parasite control is not significantly different upon further stratification by PCR status. (**a**) Stratification of TBS^−^ Africans by PCR status to TBS^−^PCR^+^ and TBS^−^PCR^−^. Created with BioRender.com. (**b**) The frequency of IFNγ^+^TNF^−^CD4^+^ T cells at baseline using the data from Fig. 1d, but only including TBS^−^ Africans, which were stratified by PCR status after PfSPZ DVI. Horizontal lines indicate the median and all data points are added. P-value from Welch’s t-test. (**c**) The frequency of IFNγ^+^TNF^+^DN^+^ γδ T at baseline using the data from Fig. 1j, but only including TBS^−^ Africans stratified by PCR. Horizontal lines indicate the median and all data points are added. P-value from Welch’s t-test. (**d**) change in the frequencies of IFNγ^+^TNF^−^CD4^+^ T cells (left panel) and IFNγ^+^TNF^+^DN^+^ γδ T cells (right panel) after PfSPZ DVI. The frequencies on five (D + 5) and eleven (D + 11) days after PfSPZ DVI were compared against the baseline using linear mixed models to obtain the estimates and corresponding 95% confidence intervals (CI). The estimates and intervals were visualized using error bars with either solid lines if statistically significant (P < 0.05) or dashed lines if otherwise. P-values for selected comparisons were shown.

A more sensitive detection by qPCR was also used to detect the emergence of very low numbers of parasites in blood. Of eight individuals who remained TBS^-^ after PfSPZ DVI, four developed submicroscopic parasitemia (TBS^−^PCR^+^) and the rest remained negative (TBS^−^PCR^−^) even by this sensitive assay (Fig. 4a, Supplementary Fig. S1b). Undetectable parasites by qPCR might indicate either sterilizing pre-erythrocytic immunity, in which malaria infection was abrogated before or during the liver stage, or strong and rapid blood-stage immunity that could get rid of the parasites. Here we explored the correlates of protective NAI that distinguish TBS^−^PCR^−^ from TBS^−^PCR^+^ individuals.

We first investigated how the frequencies of cytokine-producing cells associated with TBS^−^ Africans differ upon stratification by PCR status. The baseline frequency of IFNγ^+^CD4^+^ T was not significantly different between TBS^−^PCR^+^ and TBS^−^PCR^−^(Fig. 4b), however, it is interesting to note that the frequencies tend to be higher in TBS^−^PCR^+^ individuals. The baseline frequencies of IFNγ^+^TNF^+^ DN γδ T cells were also comparable between the groups (Fig. 4c). Following PfSPZ DVI, frequencies of both IFNγ^+^TNF^−^CD4^+^ T and IFNγ^+^TNF^+^DN γδ T cells significantly decreased only in TBS^−^PCR^-^ (Fig. 4d). However, interaction contrast comparing the change in frequencies from baseline to day five between TBS^−^PCR^+^ and TBS^−^PCR^-^ was not significantly different for either IFNγ^+^TNF^−^CD4^+^ T (estimate ± SE, -0.011 ± 0.013, P = 0.38) or IFNg^+^TNF^+^DN γδ T cells (estimate ± SE, 0.00384 ± 0.068, P = 0.95), which suggests a comparable magnitude of change in frequencies after PfSPZ DVI between both groups. Therefore, the profile of cytokine-producing cell subsets associated with TBS^−^ Africans is not significantly different upon further stratification of these individuals by their PCR status.

**Figure 5:**
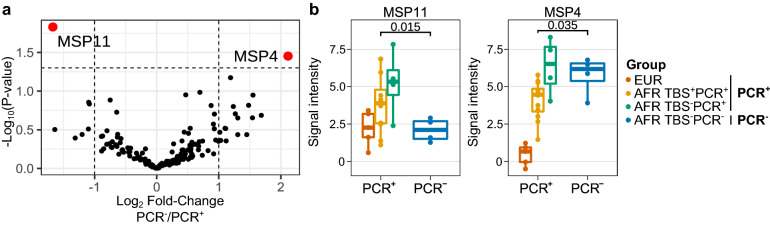
Anti-malarial antibodies associated with sub-microscopic parasitaemic individuals. (**a**) Antibody reactivity of PCR^+^ (including Europeans, TBS^+^ Africans, and TBS^−^PCR^+^ Africans) and PCR^−^ individuals to Plasmodium protein microarray, showing the reactivities at baseline (C-1) to two statistically significant antigens (P-value < 0.05 and absolute Log2 fold-change > 1, annotated). Statistics are based on two-sided Welch t-tests. (**b**) Boxplots of PCR^+^ individuals were stratified by both nationality (Europeans or Africans) and TBS status (TBS^+^ or TBS^−^). All data points are added. P-values were obtained from t-tests comparing signal intensities of PCR^+^ and PCR^−^ individuals.

Regarding the antibody data, we found that antibody reactivities against *Plasmodium* proteins that were previously found to be the highest in TBS^−^ individuals^17^ tend to be the highest in TBS^−^PCR^+^ (Supplementary Fig. S6) , not the TBS^−^PCR^−^ group. The comparison of antibody reactivity between PCR^−^ against all PCR^+^ individuals revealed MSP11 as being associated with PCR^+^ (Fig. 5a), with the highest levels observed in the TBS^−^PCR^+^ African individuals (Fig. 5b). This suggests the involvement of these antibodies in strong control of the levels of parasites in the TBS^−^PCR^+^ group that carried very low levels of parasites only detectable by the sensitive PCR test.

**Figure 6:**
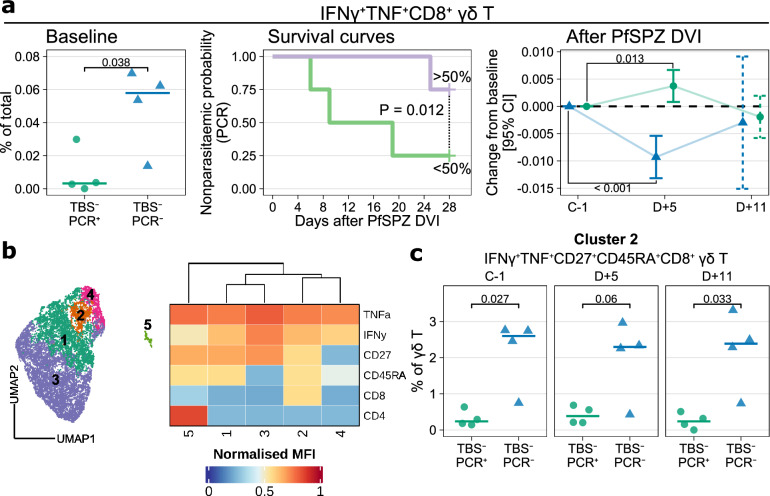
Higher baseline and early decrease in the frequencies of IFNγ^+^TNF^+^CD8^+^ γδ T cells are associated with sterilizing immunity in lifelong malaria-exposed individuals. (**a**) Profile of IFNγ^+^TNF^+^CD8^+^ γδ T cells at baseline and after PfSPZ DVI. Left panel, baseline frequencies of the subset at baseline. All data points are added, and the horizontal line represents median frequencies for each respective group. P-values from Welch’s t-test. Center panel, survival curve showing time until parasitemia according to the baseline frequency of IFNγ^+^TNF^+^CD8^+^ γδ T cells. The frequency was split on the median to create two groups of individuals, a top > 50% (purple) and a bottom < 50% (green). This grouping was then used to compare the survival distributions of the groups using the log-rank test. Right panel, changes in the frequency of IFNγ^+^TNF^+^CD8^+^ γδ T cells after PfSPZ DVI. The frequencies at D + 5 or D + 11 were compared against the baseline using linear mixed models to obtain the estimates and corresponding 95% confidence intervals (CI). The estimates and intervals were visualized using error bars with either solid lines if statistically significant (P < 0.05) or dashed lines if otherwise. (**b**) UMAP embedding of all cytokine-producing γδ T cells with an overlay of the clusters in different colors (left panel) and a heatmap summary of median expression values of markers expressed by the five γδ T cell clusters (right panel). (**f**) The frequency of γδ T cells cluster 2 (IFNγ^+^TNF^+^CD27^+^CD45RA^+^CD8^+^ γδ T cells), which was obtained from (**b**) at each time point. P-values from Welch’s t-test.

We then investigated for cell subsets associated with PCR^−^ that were not previously identified as being associated with remaining TBS-negative. We found that the baseline frequency of IFNγ^+^TNF^+^CD8^+^ γδ T cells was higher in the TBS^−^PCR^−^, P = 0.038 (Fig. 6a, left panel). Kaplan–Meier plot showed that higher frequencies of IFNγ^+^TNF^+^CD8^+^ γδ T cells was associated with a higher probability of being PCR^−^ (Fig. 6a, center panel). Following PfSPZ DVI, the frequencies of IFNγ^+^TNF^+^CD8^+^ γδ T cells significantly decreased on day five in TBS^−^PCR^−^ and contrastingly increased in TBS^−^PCR^+^ subjects (Fig. 6a, right panel). Analysis of IFNγ^+^TNF^+^CD8^+^ γδ T cells in all individuals showed no significant difference at baseline, nor a change following PfSPZ DVI in any of the groups (Supplementary Fig. S7). We then performed an unsupervised analysis of cytokine-producing γδ T cells, which revealed 5 clusters, with cluster 2 being positive for CD8 (Fig. 6b). We found that cluster 2, an IFNγ^+^TNF^+^CD8^+^ γδ T cell cluster that co-expressed CD27 and CD45RA, was significantly higher in TBS^-^PCR^-^ at baseline (P = 0.027) and eleven days after PfSPZ DVI (P = 0.033) (Fig. 6c). Thus, higher baseline frequencies and early decrease of IFNγ^+^TNF^+^CD8^+^ γδ T cells were associated with the ability to remain PCR-negative after PfSPZ DVI.

Taken together, we showed that cytokine-producing CD8^+^ γδ T cells were associated with resistance to parasitemia as detected by PCR, a highly sensitive assay, suggesting the importance of these cells in naturally acquired immunity, which acts against either the liver- or the early blood-stage parasites.

## Discussion

In this study, we have found that IFNγ-producing CD4^+^ T cells and DN γδ T cells co-producing IFNγ and TNF are associated with parasite control among lifelong malaria-exposed Africans. The functional relevance of the IFNγ-producing CD4^+^ T cells is corroborated through the significant correlations with antibody reactivities to blood-stage *Pf* proteins. Further analysis of TBS^−^ Africans who remained PCR-negative for the whole study period of 28 days revealed CD8^+^ γδ T cells co-producing IFNγ and TNF as a potential correlate of sterilizing immunity.

It is widely believed that repeated natural malaria infection could induce protection against severe disease but not sterilizing immunity against infection^29^. Following CHMI, it has been shown that naturally acquired immunity can develop against infection, which was not associated with sickle cell trait^5^. Here we analyzed samples from volunteers of a CHMI study conducted in a malaria-endemic area, which involves malaria-naïve Europeans and lifelong malaria-exposed Africans, to identify immune profiles associated with protection against malaria infection in the context of naturally acquired immunity.

Malaria-specific IFNγ^+^CD4^+^ T cells have been previously identified as a correlate of protection in the context of vaccination^30,31^ and natural infection^7^. However, stage-specificity and the precise mechanism by which IFNγ-producing CD4^+^ T cells mediate parasite control remain elusive^11^. Here we showed that higher baseline frequencies and a subsequent early decrease of malaria-specific IFNγ^+^CD4^+^ T cells following PfSPZI DVI, which precedes the first detection of malaria parasite in the blood even by PCR, were associated with control of parasitemia. Previously, we showed that the frequencies of CD161^+^CD4^+^ T cells were correlated with antibody reactivities against *Plasmodium* proteins, thus hinting at its functional relevance in protection against malaria infection^17^. Here we showed that functionally active, antigen-specific IFNγ-producing CD4^+^ T cell frequencies correlate with antibody reactivities against several *Plasmodium* proteins, including MSP4, STARP, and PF3D7_1360400, suggesting their importance in the maintenance of humoral immunity against malaria^11^. Exploration of the *Plasmodium* gene expression database shows that these genes were preferentially expressed in the asexual blood-stage parasites, which suggests the importance of these correlates in protection against blood-stage malaria. Indeed, the protective role of naturally acquired anti-MSP4 antibodies has been shown previously^32,33^. With that said, we also observed an early decrease in the frequencies of IFNγ-producing CD4^+^ T cells in TBS^−^ subjects, which could indicate migration into lymph nodes and tissues, to facilitate parasite control^34^. These data highlight the importance of both humoral and cellular immunity in providing capability to control parasite in the context of naturally acquired immunity. Taken together, we have shown the association of cytokine-producing CD4^+^ T cells in naturally acquired immunity and its correlation with anti-malarial antibodies, which might synergize to mediate the control of blood-stage malaria parasites.

Innate-like lymphocytes, which include γδ T cells, are known as the first line of defense against malaria^35^. Despite the comparable baseline frequencies of IFNγ^+^TNF^+^ DN γδ T cells among the groups, a significant decrease in frequency was observed only in TBS^−^ Africans within five days after PfSPZ DVI. As these volunteers did not develop patent parasitemia, this decrease is likely to be caused by the migration of γδ T cells out of blood, possibly into the liver, to mediate parasite killing rather than loss or antigen hypo-responsiveness of the γδ T cells^36^. A concurrent decrease in the frequency of both γδ T and CD4^+^ T cells suggests that these subsets might be part of an immune program that confers protection in tissues that participate in immunity to malaria parasites.

Sterilizing malaria immunity could be achieved in several ways: preventing hepatocyte infection by sporozoites; terminating liver-stage infection by tissue-resident cells or infiltrating immune cells, or; the immediate abrogation of merozoites upon bloodstream entry^29,37^. Of eight volunteers who remained blood-smear negative, four remained negative by PCR, a more sensitive detection method. The immunological profile of the African individuals who were TBS^−^ but PCR^+^, thus carrying low parasite densities, was characterized by relatively higher frequencies of IFNγ^+^CD4^+^ T cells and antibodies, which might be directed at blood-stage parasites. Interestingly, we observed higher levels of anti-MSP11 antibodies in PCR^+^ individuals, specifically the TBS^−^PCR^+^ Africans, compared to the PCR^−^ Africans. MSP11 has been proposed as a blood-stage vaccine candidate^38^. It is possible that TBS^-^PCR^+^ African individuals could control parasitemia to remain below microscopic detection limit owing to the high levels of anti-MSP11 antibodies.

When considering the TBS- and PCR-negative individuals, CD8^+^ γδ T cells seemed to distinguish them from the TBS^−^PCR^+^ subjects. We observed a higher frequency of IFNγ^+^TNF^+^CD8^+^ γδ T cells at baseline in TBS^−^PCR^−^ African individuals, which decreased early following PfSPZ DVI. Studies of CD8^+^γδ T cells in patients who received bone marrow stem cell grafts, revealed these cells to show higher proliferation and activation marker expression upon TCR stimulation compared to CD8^−^γδ T cells in those who experienced increased incidence of acute graft versus host disease^39^. In the context of inflammatory bowel disease, CD8^+^γδ T cells have been shown to have cytotoxic potential, able to produce IFNγ and TNF, and correlate negatively with disease activity^40^. Unbiased clustering analysis of cytokine-producing γδ T cells showed that the CD8^+^ γδ T cells co-expressed CD27, CD45RA, and co-produced IFNγ and TNF. Although CD45RA^+^CD27^+^ γδ T cells are considered naïve, their cytokine-producing capacity is not as impaired as naïve αβ T cells, when compared to their memory counterparts^41^. Moreover, CD27 has been described as a mediator of γδ T cell activation and Th1 cytokine production^42^.

Our study is limited by the relatively small sample size, which is characteristic of many controlled human infection studies. Moreover, infection by PfSPZ DVI may not mimic natural infection by mosquito bites as it bypasses the immunoregulatory environment of the skin^43^. However, we show the utility of CHMI in studying innate and adaptive cellular NAI against malaria in lifelong-malaria-exposed volunteers.

Our study highlights IFNγ^+^CD4^+^ T and γδ T cells co-producing IFNγ and TNF as well as anti-malarial antibodies against blood-stage proteins as correlates of varying strength of naturally acquired immunity in lifelong residents of a malaria-endemic area. We also showed the potential role of cytokine-producing CD8^+^ γδ T cells in conferring protection against liver-stage malaria in these subjects, which leads to sterilizing immunity. Taken together, this further highlights the complexity of naturally acquired immunity and the necessity of targeting multiple life stages of the malaria parasite to achieve strong parasite control.

## Supplementary Information

Supplementary Information.

## Data Availability

Flow cytometry data of this study are available at ImmPort under study accession SDY1734. Further information is available from the corresponding author upon reasonable request.

## References

[1] WHO (2020). World Malaria Report 2020.

[2] Sissoko MS (2017). Safety and efficacy of PfSPZ Vaccine against *Plasmodium falciparum* via direct venous inoculation in healthy malaria-exposed adults in Mali: A randomised, double-blind phase 1 trial. Lancet. Infect. Dis..

[3] Regules JA (2016). Fractional third and fourth dose of RTS, S/AS01 malaria candidate vaccine: A Phase 2A controlled human malaria parasite infection and immunogenicity study. J. Infect. Dis..

[4] Obiero JM (2015). Impact of malaria preexposure on antiparasite cellular and humoral immune responses after controlled human malaria infection. Infect. Immun..

[5] Lell B (2018). Impact of sickle cell trait and naturally acquired immunity on uncomplicated malaria after controlled human malaria infection in adults in Gabon. Am. J. Trop. Med. Hyg..

[6] Jagannathan P (2017). Vδ2+ T cell response to malaria correlates with protection from infection but is attenuated with repeated exposure. Sci. Rep..

[7] Jagannathan P (2014). IFNγ/IL-10 co-producing cells dominate the CD4 response to malaria in highly exposed children. PLoS Pathog..

[8] Stanisic DI, Good MF (2016). Examining cellular immune responses to inform development of a blood-stage malaria vaccine. Parasitology.

[9] Cohen S, McGregor IA, Carrington S (1961). Gamma-globulin and acquired immunity to human malaria. Nature.

[10] Triller G (2017). Natural parasite exposure induces protective human anti-malarial antibodies. Immunity.

[11] Kurup SP, Butler NS, Harty JT (2019). T cell-mediated immunity to malaria. Nat. Rev. Immunol..

[12] Horowitz A (2010). Cross-talk between T cells and nk cells generates rapid effector responses toplasmodium falciparum-infected erythrocytes. J. Immunol..

[13] Su Z, Stevenson MM (2000). Central role of endogenous gamma interferon in protective immunity against blood-stage Plasmodium chabaudi AS infection. Infect. Immun..

[14] Seguin MC (1994). Induction of nitric oxide synthase protects against malaria in mice exposed to irradiated *Plasmodium berghei* infected mosquitoes: Involvement of interferon gamma and CD8+ T cells. J. Exp. Med..

[15] Zaidi I (2017). γδ T cells are required for the induction of sterile immunity during irradiated sporozoite vaccinations. J. Immunol..

[16] Troye-Blomberg M (1999). Human γδT cells that inhibit the in vitro growth of the asexual blood stages of the *Plasmodium falciparum* parasite express cytolytic and proinflammatory molecules. Scand. J. Immunol..

[17] De Jong SE (2021). Systems analysis and controlled malaria infection in Europeans and Africans elucidate naturally acquired immunity. Nat. Immunol..

[18] Hoffman SL (2010). Development of a metabolically active, non-replicating sporozoite vaccine to preventPlasmodium falciparummalaria. Hum. Vaccines.

[19] Teirlinck AC (2011). Longevity and composition of cellular immune responses following experimental Plasmodium falciparum malaria infection in humans. PLoS Pathog..

[20] Hahne F (2009). flowCore: A Bioconductor package for high throughput flow cytometry. BMC Bioinform..

[21] McInnes L, Healy J, Saul N, Großberger L (2018). UMAP: Uniform manifold approximation and projection. J. Open Source Softw..

[22] Gu Z, Eils R, Schlesner M (2016). Complex heatmaps reveal patterns and correlations in multidimensional genomic data. Bioinformatics.

[23] Borrmann S (2020). Mapping of Safe and Early Chemo-attenuated Live Plasmodium falciparum Immunization Identifies Immune Signature of Vaccine Efficacy.

[24] Sulyok Z (2021). Heterologous protection against malaria by a simple chemoattenuated PfSPZ vaccine regimen in a randomized trial. Nat. Commun..

[25] Silver JD, Ritchie ME, Smyth GK (2009). Microarray background correction: Maximum likelihood estimation for the normal-exponential convolution. Biostatistics.

[26] Aurrecoechea C (2009). PlasmoDB: A functional genomic database for malaria parasites. Nucleic Acids Res..

[27] R Core Team. *R: A Language and Environment for Statistical Computing v. 4.0.2* (R Foundation for Statistical Computing, 2020).

[28] Wickham H (2019). Welcome to the Tidyverse. J. Open Source Softw..

[29] Doolan DL, DobañO C, Baird JK (2009). Acquired immunity to malaria. Clin. Microbiol. Rev..

[30] Ishizuka AS (2016). Protection against malaria at 1 year and immune correlates following PfSPZ vaccination. Nat. Med..

[31] Moncunill G (2017). RTS, S/AS01E malaria vaccine induces memory and polyfunctional T cell responses in a pediatric African Phase III Trial. Front. Immunol..

[32] Perraut R (2017). Association of antibodies to Plasmodium falciparum merozoite surface protein-4 with protection against clinical malaria. Vaccine.

[33] Naung MT (2022). Global diversity and balancing selection of 23 leading Plasmodium falciparum candidate vaccine antigens. PLoS Comput. Biol..

[34] Crompton PD (2014). Malaria immunity in man and mosquito: Insights into unsolved mysteries of a deadly infectious disease. Annu. Rev. Immunol..

[35] Deroost K, Langhorne J (2018). Gamma/delta T cells and their role in protection against malaria. Front. Immunol..

[36] Jagannathan P (2014). Loss and dysfunction of Vδ2^+^ γδ T cells are associated with clinical tolerance to malaria. Sci. Transl. Med..

[37] Langhorne J, Ndungu FM, Sponaas A-M, Marsh K (2008). Immunity to malaria: More questions than answers. Nat. Immunol..

[38] Tohmoto T (2019). Anti-MSP11 IgG inhibits Plasmodium falciparum merozoite invasion into erythrocytes in vitro. Parasitol. Int..

[39] Gaballa A, Arruda LCM, Rådestad E, Uhlin M (2019). CD8<sup>+</sup><i>γδ</i> T cells are more frequent in CMV seropositive bone marrow grafts and display phenotype of an adaptive immune response. Stem Cells Int..

[40] Kadivar M, Petersson J, Svensson L, Marsal J (2016). CD8αβ^+^ γδ T Cells: A novel T cell subset with a potential role in inflammatory bowel disease. J. Immunol..

[41] Xu W (2019). Mapping of γ/δ T cells reveals Vδ2+ T cells resistance to senescence. EBioMedicine.

[42] Debarros A, Chaves-Ferreira M, D'Orey F, Ribot JC, Silva-Santos B (2011). CD70-CD27 interactions provide survival and proliferative signals that regulate T cell receptor-driven activation of human γδ peripheral blood lymphocytes. Eur. J. Immunol..

[43] Honda T, Miyachi Y, Kabashima K (2011). Regulatory T cells in cutaneous immune responses. J. Dermatol. Sci..

