# Supplementary material for: Immunological profiles associated with distinct parasitemic states in volunteers undergoing malaria challenge in Gabon
Source: Sci Rep. 2022 Aug 3;12:13303. doi: 10.1038/s41598-022-17725-8 (PMC9349185; doi:10.1038/s41598-022-17725-8)
Supplement: Supplementary file 1 — Supplementary Information. [file 41598_2022_17725_MOESM1_ESM.pdf]

Supplementary information

Immunological profiles associated with distinct parasitaemic states in volunteers undergoing malaria challenge in Gabon

Mikhael D. Manurung*[1], Sanne E. de Jong[1], Yvonne Kruize[1], Yoanne D. Mouwenda[1,2], Madeleine Eunice Betouke Ongwe[1,2,3], Yabo Josiane Honkpehedji[1,2], Jeannot Frézus Zinsou[2,4], Jean Claude Dejon-Agobe[2,5], Stephen L. Hoffman[6], Peter G. Kremsner[2,7,8], Ayola Akim Adegnika[1,2,4,7,8], Rolf Fendel[2,7,8], Benjamin Mordmüller[2,7,8#], Meta Roestenberg[1], Bertrand Lell[2,9], Maria Yazdanbakhsh[1]

Supplementary Table S1. Demographics and parasitological outcome of the CHMI trial volunteers.

Characteristic	Europeans	Africans	
		TBS$^+$	TBS$^-$
n	5	12	8
Sex, male (n, %)	1 (20%)	8 (66.7%)	4 (50%)
HbAA haemoglobin phenotype	5 (100%)	7 (58.3%)	4 (50%)
Age, years	28.0 (24.3-28.8)	22.4 (19.0-25.9)	21.6 (18.6-26.6)
BMI, kg/m^2	23.3 (18.9-26.6)	22.6 (16.2-27.0)	19.9 (19.2-22.9)
Parasitaemic by PCR (n, %)	5 (100%)	12 (100%)	4 (50%)
Days to parasitaemia by PCR (GM, range)	7.9 (7-9)	8.1 (6-11)	17.4 (6-NA[a])
Parasitaemic by TBS (n, %)	5 (100%)	12 (100%)	0 (0%)
Days to parasitaemia by TBS (GM, range)	12.4 (12-14)	17.9 (13-25)	-

All values are number (%) or median (range) unless mentioned otherwise. [a]All volunteers were treated on Day 28. PCR, polymerase chain reaction. GM, geometric mean.

Supplementary Table S2. Materials and reagents used in this study.

Materials and Reagents	Manufacturer	Cat#	End concentration (if applicable)
**CS&T Research Beads**	BD Biosciences	655050	
**Formaldehyde solution 37%**	Sigma-Aldrich	F1635	1.9%
**eBioscience™ Permeabilization Buffer (10X)**	ThermoFisher	00-8333-56	1:10 in milliQ
**Anti-Mouse Ig, κ/Negative Control Compensation Particles Set**	BD Biosciences	552843	
**Anti-Rat and Anti-Hamster Ig κ /Negative Control Compensation Particles Set**	BD Biosciences	552845	
**ArC™ Amine Reactive Compensation Bead Kit**	ThermoFisher	A10346	
**Human FcR binding inhibitor purified**	eBiosciences	14-9161-73	1:100
**RPMI-1640 Medium**	ThermoFisher	22409-015	
**Hank's balanced salt solution**	ThermoFisher	24020117	
**Uninfected erythrocytes**	*In-house reagent*	N/A	1:1 with cells
***P. falciparum*-infected erythrocytes**	*In-house reagent*	N/A	1:1 with cells
**Staphylococcal enterotoxin B**	Sigma	S4881	200 ng/mL
**Brefeldin A**	Sigma	B7651	10 μg/mL
**Non-sterile 96-wells V-bottom plate**	Nunc	249570	
**Sterile 96-wells round-bottom plate**	Corning	3799	
**1.4 mL FACS insert tubes**	Micronic	MP32022	
**Foetal bovine serum (FBS)**	Greiner Bio-one	75809356165	

Supplementary Table S3. Flow cytometry antibodies.

Specificity	Clone	Fluorochrome	Manufacturer	Cat#
**CD3**	SK7	PerCP	BioLegend	344813
**CD4**	3G8	PE-DZ594	BioLegend	302054
**CD8**	RPA-T8	AF-700	BioLegend	301027
**TCRγδ**	11F2	FITC	BD Biosciences	347903
**CD56**	NCAM 16.2	BV711	BD Biosciences	563169
**CD14**	M5E2	BV650	BioLegend	301836
**CD45RA**	HI100	APC-ef780	BioLegend	304127
**CD27**	L128	BV786	BioLegend	563327
**IFNγ**	4S.B3	PE	BioLegend	502509
**TNF**	MAb11	PE-Cy7	BioLegend	502929
**IL-2**	MQ1-17H12	BV605	BioLegend	500332
**IL-17**	BL168	APC	BioLegend	512334
**PD-1**	EH12.2H7	BV421	BioLegend	329920
**Viability**	N/A	LIVE/DEAD™ Fixable Aqua	ThermoFisher	L34957

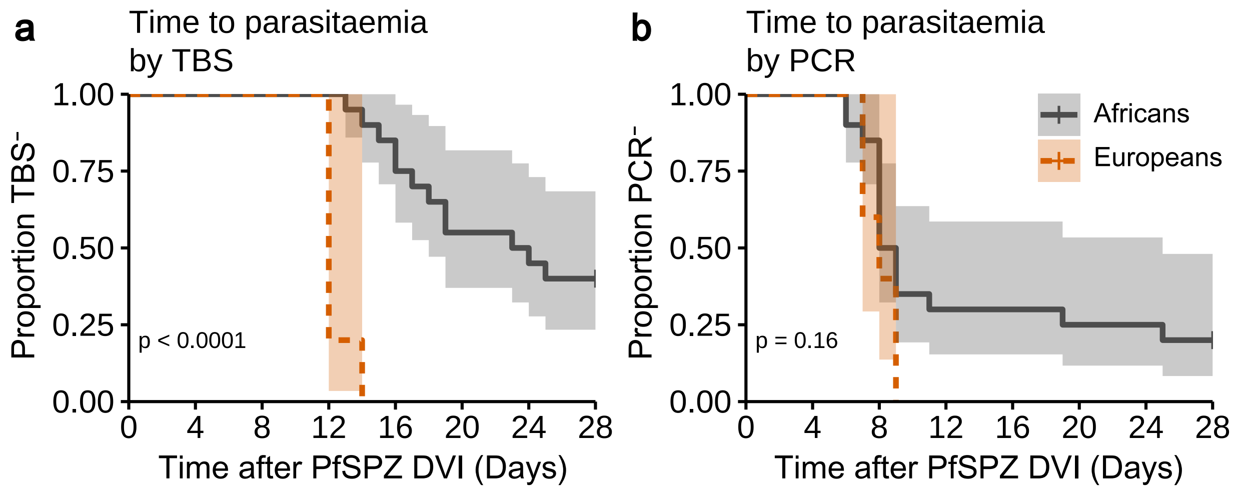

Fig. S1. Kaplan-Meier plots of time to parasitemia after PfSPZ DVI in all European and African individuals as defined by TBS **(a)** or PCR **(b)**. P-values from the log-rank test comparing the two curves.

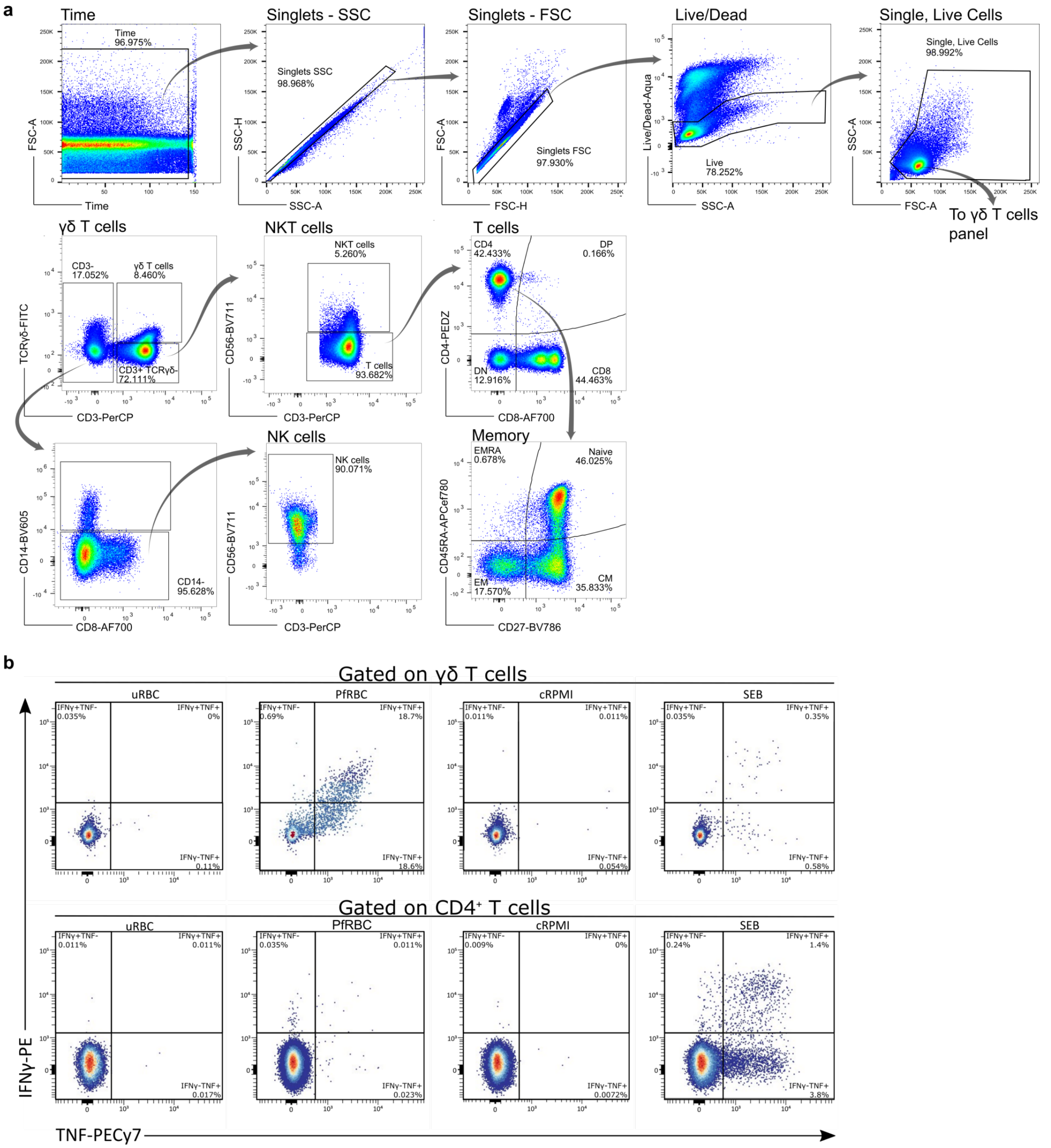

Fig. S2. Flow cytometry gating strategy. a, Representative flow cytometry plots for the gating of immune cell subsets after quality control gating for time, singlets, and live cells. **b**, Gating strategy of IFNγ and TNF with examples for γδ T and CD4⁺ T cells from one of the malaria lifelong-exposed individuals. The gating was initially set on the uRBC control and then applied to the samples stimulated with PfRBC. The negative (medium; cRPMI) and positive (SEB) controls were used as references.

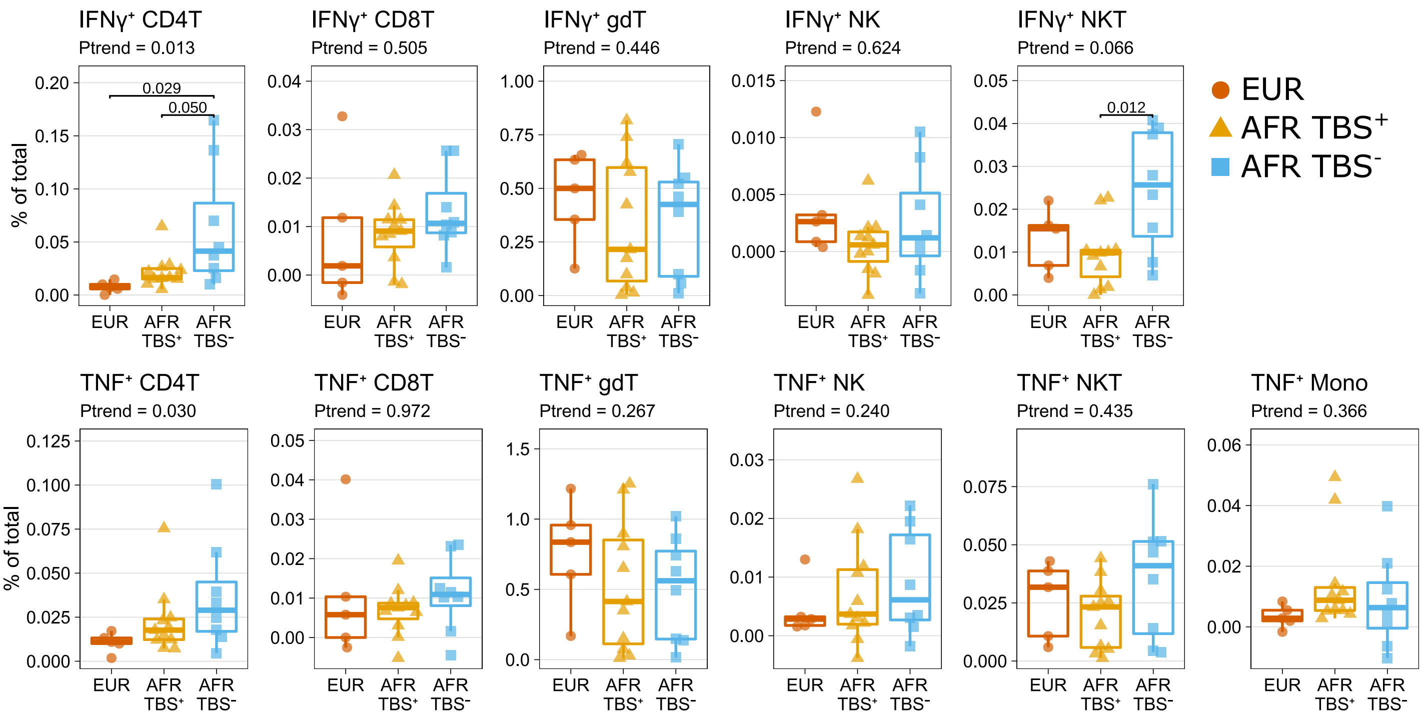

Fig. S3. Baseline frequencies of cells producing IFNγ or TNF as shown in Fig. 1b for every subsets. The boxplots show the median, 1st, and 3rd quartiles and the whiskers extend to the maximum/minimum of the respective groups, no further than 1.5x the IQR. All data points are added. P-values of the pairwise comparisons were obtained using Tukey's honestly significant difference (HSD) test. P-values of linear trend (P_{trend}) in increase/decrease of frequencies from Europeans, TBS^+ to TBS^- Africans were obtained using linear models with orthogonal polynomial contrasts. Note that IFNγ was not analysed for monocytes.

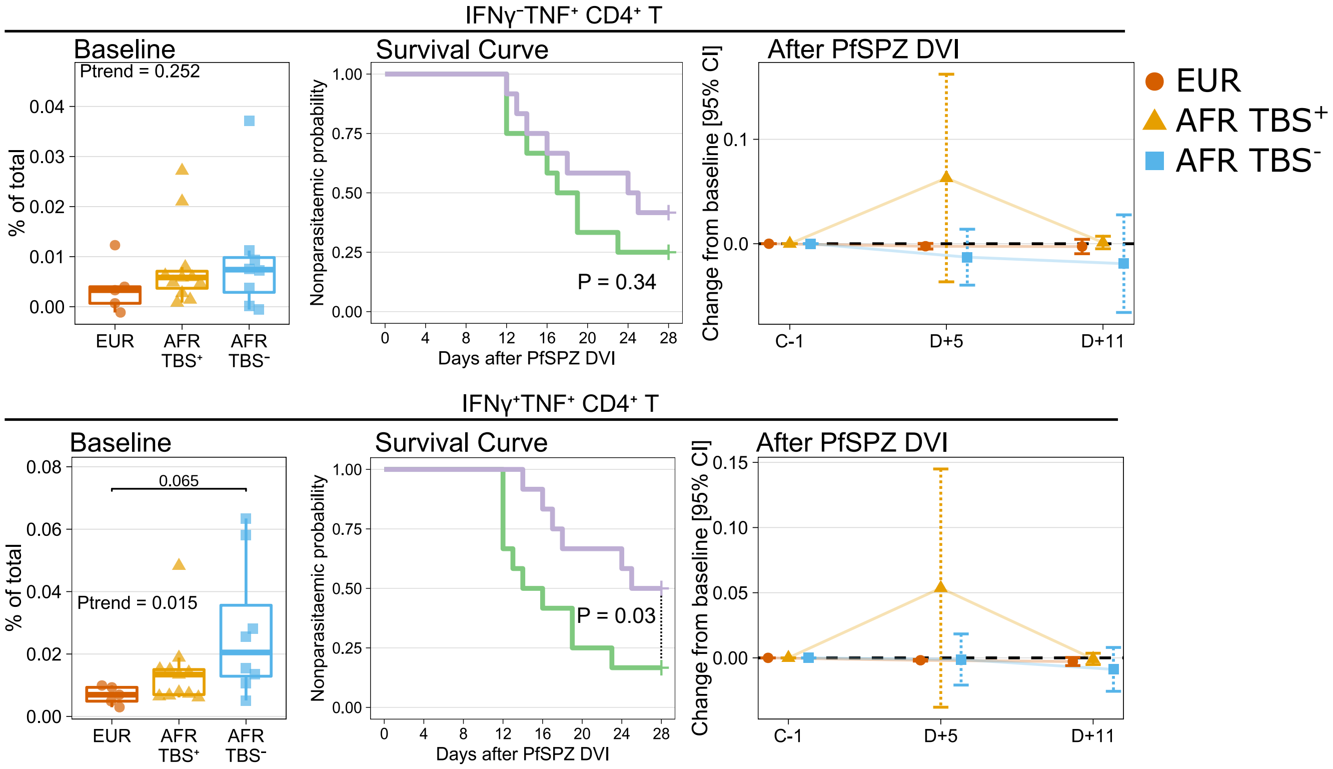

Fig. S4. Frequencies of IFNγ⁻TNF⁺ (top) and IFNγ⁺TNF⁺ (bottom) CD4⁺ T cells at baseline and after PfSPZ DVI. Left panel, baseline frequencies of cytokine-producing cells. The boxplots show the median, 1st, and 3rd quartiles and the whiskers extend to the maximum/minimum of the respective groups, no further than 1.5x the IQR. All data points are added. P-values of the pairwise comparisons were obtained using Tukey's honestly significant difference (HSD) test. P-values of linear trend (Ptrend) in increase/decrease of frequencies from Europeans, TBS⁺ to TBS⁻ Africans were obtained using linear models with orthogonal polynomial contrasts. **Center panel**, survival curves showing time to parasitaemia according to the baseline frequency of the respective cytokine-producing subset. The frequency was split on the median to create two groups of individuals, a top >50% (purple) and a bottom 50% (green). This grouping was then used to compare the survival distributions of the groups using the log-rank test. **Right panel**, change in the frequencies of cytokine-producing cells after PfSPZ DVI. The frequencies on five (D+5) and eleven (D+11) days after PfSPZ DVI were compared against the baseline using linear mixed models to obtain the estimates and corresponding 95% confidence intervals (CI). The estimates and intervals were visualized using error bars with either solid lines if statistically significant ($P < 0.05$) or dashed lines if otherwise.

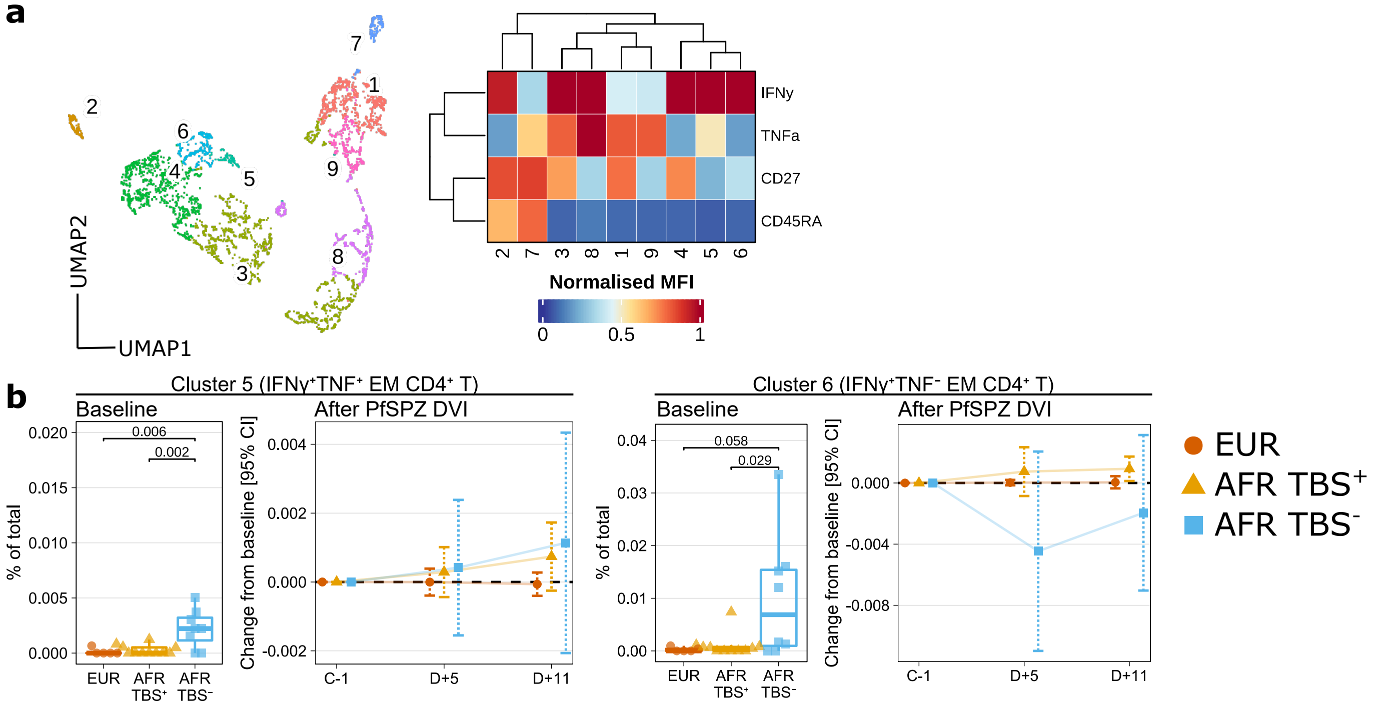

Fig. S5. Unsupervised clustering and dimensionality reduction of cytokine-producing CD4⁺ T cells. a, UMAP embedding of cytokine-producing CD4⁺ T cells with an overlay of the clusters in different colors and a heatmap summary of median expression values of markers expressed by the nine CD4⁺ T cell clusters. **b**, Baseline frequencies (left panel) and change after PfSPZ DVI (right panel) for clusters 5 and 6 obtained from **(a)**.The boxplots show the median, 1st, and 3rd quartiles and the whiskers extend to the maximum/minimum of the respective groups, no further than 1.5x the IQR. All data points are added. P-values of the baseline pairwise comparisons were obtained using Tukey's honestly significant difference (HSD) test. P-values of baseline linear trend (Ptrend) in increase/decrease of frequencies from Europeans, TBS⁺ to TBS⁻ Africans were obtained using linear models with orthogonal polynomial contrasts. The frequencies on five (D+5) and eleven (D+11) days after PfSPZ DVI were compared against the baseline using linear mixed models to obtain the estimates and corresponding 95% confidence intervals (CI). The estimates and intervals were visualized using error bars with either solid lines if statistically significant ($P < 0.05$) or dashed lines if otherwise.

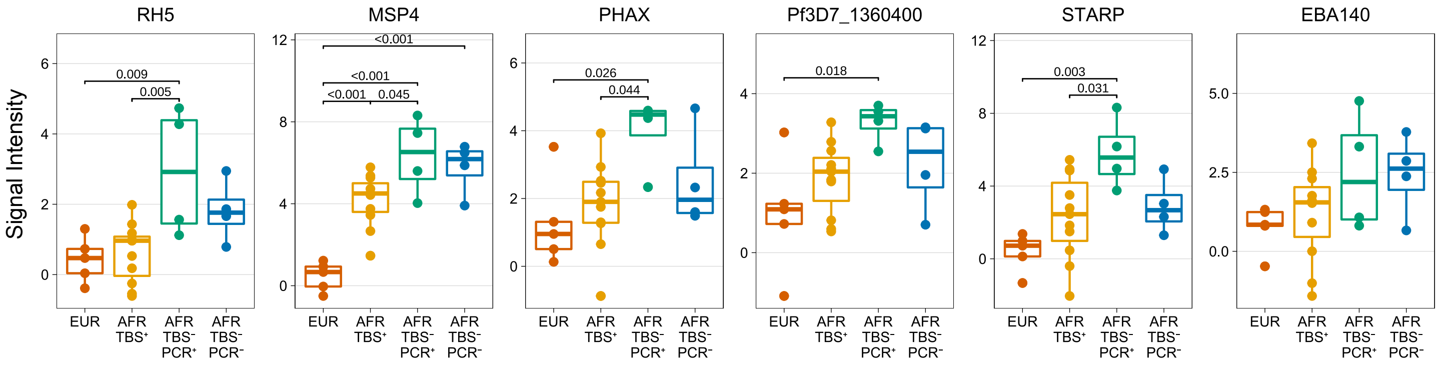

Fig. S6. Antibody reactivity to Plasmodium protein microarray showing the reactivities at baseline to six proteins associated with parasite control. Boxplots show median, 1st, and 3rd quartiles and the whiskers extend to the maximum/minimum of the respective group, no further than 1.5x the IQR. All data points are added. P-values of the pairwise comparisons were obtained using Tukey's honestly signifsicant difference (HSD) test.

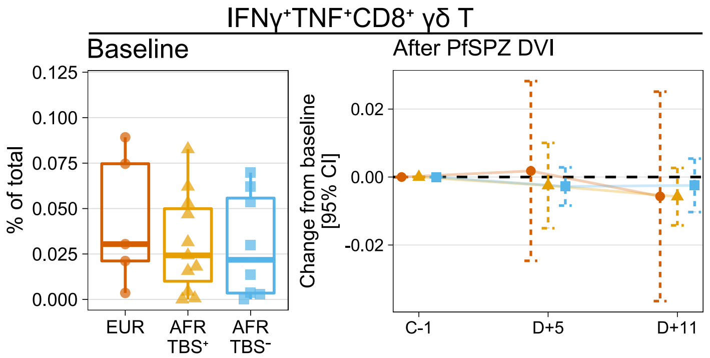

Fig. S7. Baseline frequencies (left panel) and change after PfSPZ DVI (right panel) for IFNγ⁺TNF⁺CD8⁺ γδ T cells. The boxplots show the median, 1st, and 3rd quartiles and the whiskers extend to the maximum/minimum of the respective groups, no further than 1.5x the IQR. All data points are added. P-values of the baseline pairwise comparisons were obtained using Tukey's honestly significant difference (HSD) test. The frequencies on five (D+5) and eleven (D+11) days after PfSPZ DVI were compared against the baseline using linear mixed models to obtain the estimates and corresponding 95% confidence intervals (CI). The estimates and intervals were visualized using error bars with either solid lines if statistically significant ($P < 0.05$) or dashed lines if otherwise.
